# Supplementary material for: Automated markerless pose estimation in freely moving macaques with OpenMonkeyStudio
Source: Nat Commun. 2020 Sep 11;11:4560. doi: 10.1038/s41467-020-18441-5 (PMC7486906; doi:10.1038/s41467-020-18441-5)
Supplement: Supplementary file 2 — Reporting Summary [file 41467_2020_18441_MOESM2_ESM.pdf]

nature research

Corresponding author(s): Benjamin Y. Hayden

Last updated by author(s): Jun 30, 2020

Reporting Summary

Nature Research wishes to improve the reproducibility of the work that we publish. This form provides structure for consistency and transparency in reporting. For further information on Nature Research policies, see Authors & Referees and the Editorial Policy Checklist .

Statistics

For all statistical analyses, confirm that the following items are present in the figure legend, table legend, main text, or Methods section.

n/a	Confirmed	
☐	☒	The exact sample size (*n*) for each experimental group/condition, given as a discrete number and unit of measurement
☐	☒	A statement on whether measurements were taken from distinct samples or whether the same sample was measured repeatedly
☐	☒	The statistical test(s) used AND whether they are one- or two-sided<br>*Only common tests should be described solely by name; describe more complex techniques in the Methods section.*
☒	☐	A description of all covariates tested
☐	☒	A description of any assumptions or corrections, such as tests of normality and adjustment for multiple comparisons
☐	☒	A full description of the statistical parameters including central tendency (e.g. means) or other basic estimates (e.g. regression coefficient) AND variation (e.g. standard deviation) or associated estimates of uncertainty (e.g. confidence intervals)
☒	☐	For null hypothesis testing, the test statistic (e.g. *F*, *t*, *r*) with confidence intervals, effect sizes, degrees of freedom and *P* value noted<br>*Give P values as exact values whenever suitable.*
☒	☐	For Bayesian analysis, information on the choice of priors and Markov chain Monte Carlo settings
☒	☐	For hierarchical and complex designs, identification of the appropriate level for tests and full reporting of outcomes
☒	☐	Estimates of effect sizes (e.g. Cohen's *d*, Pearson's *r*), indicating how they were calculated

Our web collection on statistics for biologists contains articles on many of the points above.

Software and code

Policy information about availability of computer code

Data collection	Data was collected with custom routines written in C++, bash and Python. Our framework relies on code from the Aravis Project (0.6.x) OpenCV (4.2.0) as well as the Spinnaker SDK (1.27.0.48).
Data analysis	Calibration routines were used from the COLMAP (3.6) project. Data was analyzed using custom routines primarily written in Python (3.6.9), Matlab (2018b) and bash. Our primary framework relies on routines for image processing in OpenCV (4.2.0). Our framework relies on package code from scipy (1.4.1), numpy (1.17.3), pyopengl (3.1.5) and pygame (1.9.6). Our deep learning pipeline is based on TensorFlow (>2.x.x).

For manuscripts utilizing custom algorithms or software that are central to the research but not yet described in published literature, software must be made available to editors/reviewers. We strongly encourage code deposition in a community repository (e.g. GitHub). See the Nature Research guidelines for submitting code & software for further information.

Data

Policy information about availability of data

All manuscripts must include a data availability statement. This statement should provide the following information, where applicable:

- Accession codes, unique identifiers, or web links for publicly available datasets
- A list of figures that have associated raw data
- A description of any restrictions on data availability

The full training dataset and model will be made available at the time of publication. The full reconstruction pipeline will be available at www.zimmermanlab.com and https://github.com/OpenMonkeyStudio

Field-specific reporting

Please select the one below that is the best fit for your research. If you are not sure, read the appropriate sections before making your selection.

☐ Life sciences ☒ Behavioural & social sciences ☐ Ecological, evolutionary & environmental sciences

For a reference copy of the document with all sections, see nature.com/documents/nr-reporting-summary-flat.pdf

Behavioural & social sciences study design

All studies must disclose on these points even when the disclosure is negative.

Study description	Computational pose estimation of non human primates using deep neural nets.
Research sample	Four representative male rhesus macaques (6-11 years old) served as subjects for the experiment. All four subjects were fed ad libitum and pair housed within a light and temperature controlled colony room. Subjects were water restricted to 25 mL/kg for initial training, and readily worked to maintain 50 mL/kg throughout experimental testing. Three of the subjects had previously served as subjects on standard neuroeconomic tasks, including a set shifting task and several simple choice tasks. Training also included experience with foraging tasks including one study using the large cage apparatus. One subject was naive to all experimental procedures. This sample represents the typical range of appearance in studies of non human primates
Sampling strategy	We chose subjects available within our colony and covered a diverse age range to obtain diverse movement patterns.
Data collection	The instrument used is our open cage design consisting of 62 machine vision cameras enclosing a large arena. Subjects were alone within the large enclosure as well as the enclosure room during data acquisition and monitored via live video feed. Experimenters were not blinded to the experimental conditions as is typical in observational studies
Timing	Data was collected between March and September 2019.
Data exclusions	No data was excluded.
Non-participation	All animals were collaborative
Randomization	Because this study is observational no experimental conditions were present

Reporting for specific materials, systems and methods

We require information from authors about some types of materials, experimental systems and methods used in many studies. Here, indicate whether each material, system or method listed is relevant to your study. If you are not sure if a list item applies to your research, read the appropriate section before selecting a response.

Materials & experimental systems

n/a	Involved in the study	
☒	☐	Antibodies
☒	☐	Eukaryotic cell lines
☒	☐	Palaeontology
☐	☒	Animals and other organisms
☒	☐	Human research participants
☒	☐	Clinical data

Methods

n/a	Involved in the study	
☒	☐	ChIP-seq
☒	☐	Flow cytometry
☒	☐	MRI-based neuroimaging

Animals and other organisms

Policy information about studies involving animals; ARRIVE guidelines recommended for reporting animal research

Laboratory animals	4 macaca mullata (5.5 - 12 kg, all male, age 5-12)
Wild animals	No wild animals were used in this study.
Field-collected samples	No field collected samples were used in this study.
Ethics oversight	University of Minnesota Institutional Animal care and Use Committee in accordance with NIH guidelines.

Note that full information on the approval of the study protocol must also be provided in the manuscript.
